# Differently expressed microRNA in response to the first Ig replacement therapy in common variable immunodeficiency patients

**DOI:** 10.1038/s41598-020-77100-3

**Published:** 2020-12-08

**Authors:** Bruna De Felice, Ersilia Nigro, Rita Polito, Francesca Wanda Rossi, Antonio Pecoraro, Giuseppe Spadaro, Aurora Daniele

**Affiliations:** 1 Dipartimento di Scienze e Tecnologie Ambientali, Biologiche e Farmaceutiche (DISTABIF), Università della Campania “Luigi Vanvitelli”, Via Vivaldi 43, 81100 Caserta, Italy; 2 CEINGE-Biotecnologie Avanzate Scarl, Via G. Salvatore 486, 80145 Napoli, Italy; 3 Dipartmento di Scienze Mediche e Chirurgiche Avanzate, Università della Campania “Luigi Vanvitelli”, Naples, Italy; 4 U.O. di Immunologia Clinica ed Allergologia-Dipartimento di Scienze Mediche E, Medicina Traslazionale Azienda Ospedaliera Universitaria, “Federico II”, Via S. Pansini 5, 80131 Napoli, Italy; 5 Department of Translational Medical Sciences (DiSMeT), Naples, Italy

**Keywords:** Genetics, Immunology, Medical research

## Abstract

Common variable immunodeficiency (CVID) is a complex primary immunodeficiency disorder characterized by a high clinical and genetic heterogeneity. The molecular underlying causes of CVID are not still now clear and the delays in diagnosis and treatment worsen the prognosis of the patients. MicroRNAs are non-coding, endogenous small RNAs often deregulated in human diseases, such as autoimmune and other immune-based disorders. In the present study, we aimed to evaluate miRNAs associated with the CVID and, in particular, with the response to the first Ig replacement therapy. To this aim, we compared miRNA profile obtained by serum samples of treatment-naïve CVID patients before and 24 h after the first Ig replacement therapy. For the first time, using a microarray assay followed by an integrated bioinformatics/biostatistics analysis, we identified five microRNAs (hsa-miR-6742, hsa-miR-1825, hsa-miR-4769-3p, hsa-miR-1228-3p, hsa-miR-1972) differently modulated in CVID patients by Ig infusion. All of them were down-regulated, excepted miR-6742 which was up-regulated. The latter may be of particular interest, since its functions are related to pathways involving Class I MHC mediated antigen processing and adaptive as well as innate Immune System. In conclusion, this study shows for the first time the modulation of miRNAs involved in CVID patients after the first Ig replacement therapy. Further studies are needed to assess whether such miRNAs could represent novel potential biomarkers in management and therapy of CVID patients.

## Introduction

Common variable immunodeficiency (CVID) is one of the most prevalent Primary Antibody Disorders characterized by marked hypogammaglobulinemia^1^. The real incidence of CVID is not simple to ascertain, but the estimated prevalence of CVID in Caucasians ranges between 1:10.000 and 1:50.000 and the prevalence rate may continue to increase^1^.

CVID is a complex heterogeneous disease characterized by deficiencies in immunoglobulin (Ig) quantity and quality, normal or decreased B-cell counts, and lack of response to protein or polysaccharide antigens^2^. The phenotypes of patients are highly heterogeneous due to different time onsets and to a high variety of related complications^2^.

The standard CVID therapy is the replacement administration of IgG as it reduces the frequency and severity of infections. However, there are some limitations of IgG replacement therapy such as the absence of treatment for non-infectious complications^3^. In addition, cancer mortality rates of CVID patients have not changed after IgG replacement therapy^2^.

Various studies have been devoted to characterize the cytokines profile in CVID, albeit with conflicting results^4–6^. We recently reported that adiponectin is decreased in CVID and correlated to the first Ig infusion, representing a serum biomarker of functional changes taking place in the adipose and related to the replacement therapy^7^.

The patients affected by CVID are characterized by highly heterogeneous and variable clinical conditions. The underlying causes of CVID in the majority of patients are still unknown, but it is likely that in the development and establishment of the disease^7^ the environmental factors have a decisive role also via epigenetic mechanisms^8^. On the other hand, the genetic influences in CVID are believed to be mutations in genes involved in the development and function of immune B cells (…). At least 13 genes have been associated with CVID, but the most frequent mutations occur in the TNFRSF13B gene that plays a role in the survival and maturation and in the production of antibodies from of B cells leading to immune dysfunction^2^. However, despite an extensive genetic analysis, most patients do not have a monogenetic diagnosis and therefore additional biological alterations participate (are at the basis) of the etiopathogenesis of the disease (Front Immunol. 2019; 10: 2678. Ameratunga). On the other hand, in the last decades, emerging evidence has demonstrated that miRNAs take part in many biological processes among which the immune functions (MicroRNAs: new regulators of immune cell development and function. D Baltimore). Indeed, modulation of miRNAs was observed in the B-cells and T-cells activation, differentiation and homeostasis, cellular processes that are important for the immune response^9^.

Delays in diagnosis and treatment worsen the prognosis of CVID patients lead to permanent organ structural damage^1–3^. Although few studies reported miRNAs regulation in response to Ig replacement therapy in immunodeficiencies, to our knowledge, there are no studies about miRNAs dosage and changes in CVID patients^10–12^.

Taking into account these observations, we evaluated the potential different regulation of miRNAs in CVID naïve-treatment patients after the first Ig replacement therapy to find new potential biomarker for CVID therapy. To this aim, have been recruited nine CVID naïve-treatment patients from which serum samples were obtained before and after the first Ig replacement therapy. The microRNA expression profile was performed by high-throughput microarray followed by extensive reverse transcription quantitative real-time PCR (RT-qPCR) validation. We identified and compared the serum miRNA pool profile of CVID patients before and after the first Ig replacement therapy.

## Results

### Anthropometric and laboratory investigations

The anthropometric and biochemical parameters of CVID naïve patients are shown in Table 1. IgG, IgM and IgA levels have been measured before and after the first Ig replacement therapy: the increase of levels of IgG is statistically significant as previously reported in patients with common variable immunodeficiencies and patients with X-linked agammaglobulinemia^13^ (Table 1). To verify the specific changes that taken place after the first IgG infusion therapy, we measured Adiponectin after 24 h as we previously reported that modification in the adiponectin levels are already evident at 24 h post-infusion and therefore we considered adiponectin as a marker of functional changes post-infusion^5,6^. We confirmed that adiponectin but not leptin was statistically different at T0 and T1 post- IgG infusion (4.3 ± 4.53 vs 12.84 ± 8.78, respectively, p < 0.01).

**Table 1:** Biochemical and anthropometrical data of CVID patients before (T0) and after (T24) the first replacement therapy with Ig infusion.

	CVID Naive Patients	T24 h post-Ig treatment	p-value
Sex	M/F = 6/3		
Body Mass Index (kg/m^2^)	24.08 ± 4.41		
IgG (mg/dl)	1.25 ± 1.38	6.92 ± 1.43	*2.4E-7*
IgA (mg/dl)	0.089 ± 0.07	0.063 ± 0.00	0.57
IgM (mg/dl)	0.09 ± 0.06	0.14 ± 0.13	0.32
Iron (µg/dl)	52.75 ± 20.07		
Glycemia (mg/dl)	77.71 ± 10.75		
Albumin (g/L)	4,2 ± 0.31		
Total Proteins (g/dl)	5.83 ± 0.54		
Fibrinogen (mg/dl)	301.5 ± 66.44		
C-reactive protein (mg/dl)	3.35 ± 1.87		
ESR (mm)	6 ± 4.83		
Adiponectin (µg/ml)	4.3 ± 4.53	12.84 ± 8.78	< 0.01

### MicroRNAs distribution in microarray libraries from CVID patients’ sera

**Figure 1:**
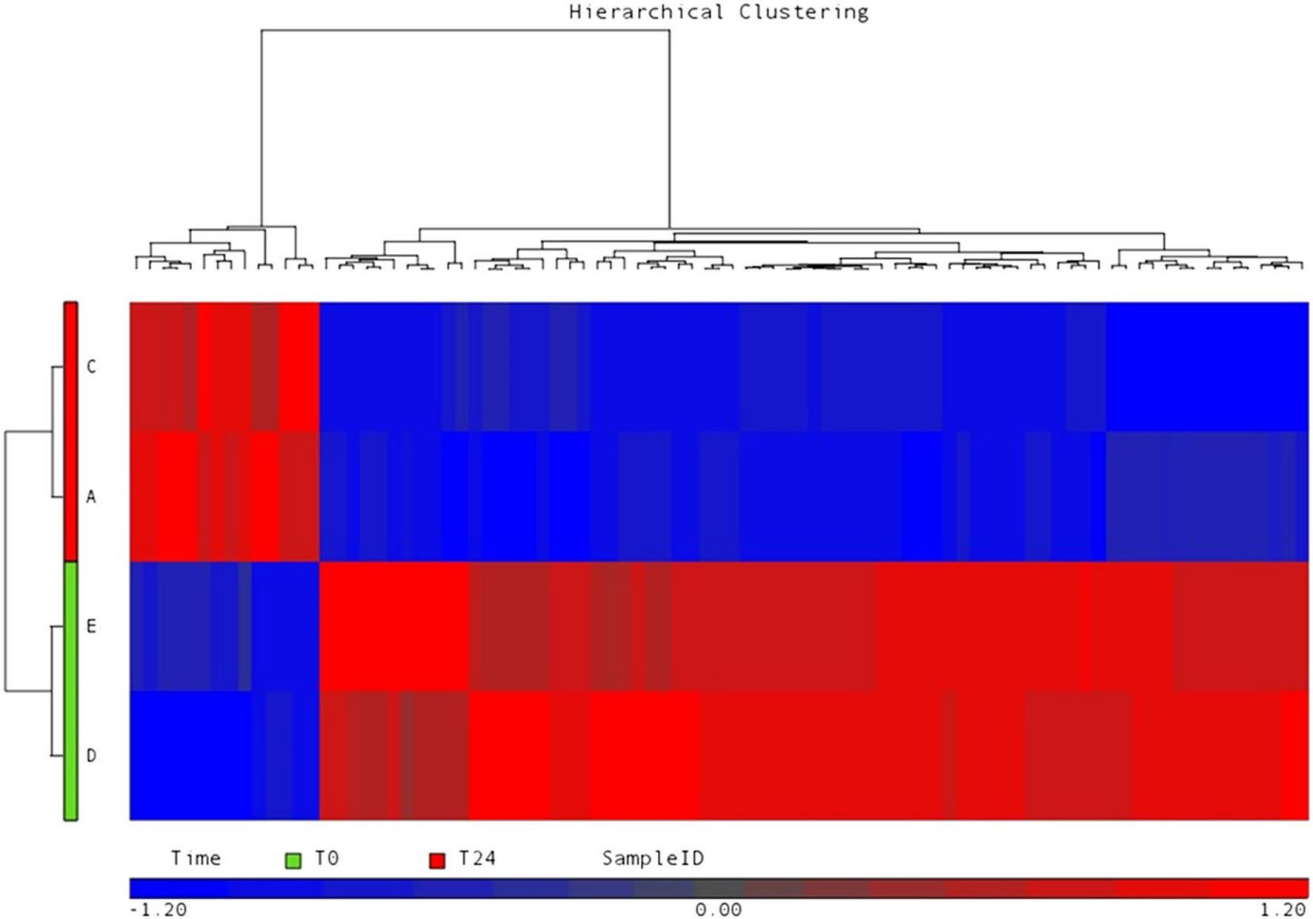
microRNA expression heat map. Differential miRNA expression in CVID serum patients after the first Ig replacement therapy (t24).The heat-map showing the differential expression pattern of miRNAs compared to CVID serum patients before the first Ig replacement therapy (t0). Blue represents low expression and red high expression. Transcriptome Analysis Console (TAC) Software from Affymetrix was used with filtering fold change = 1.5 and p-value = 0.05.

**Figure 2:**
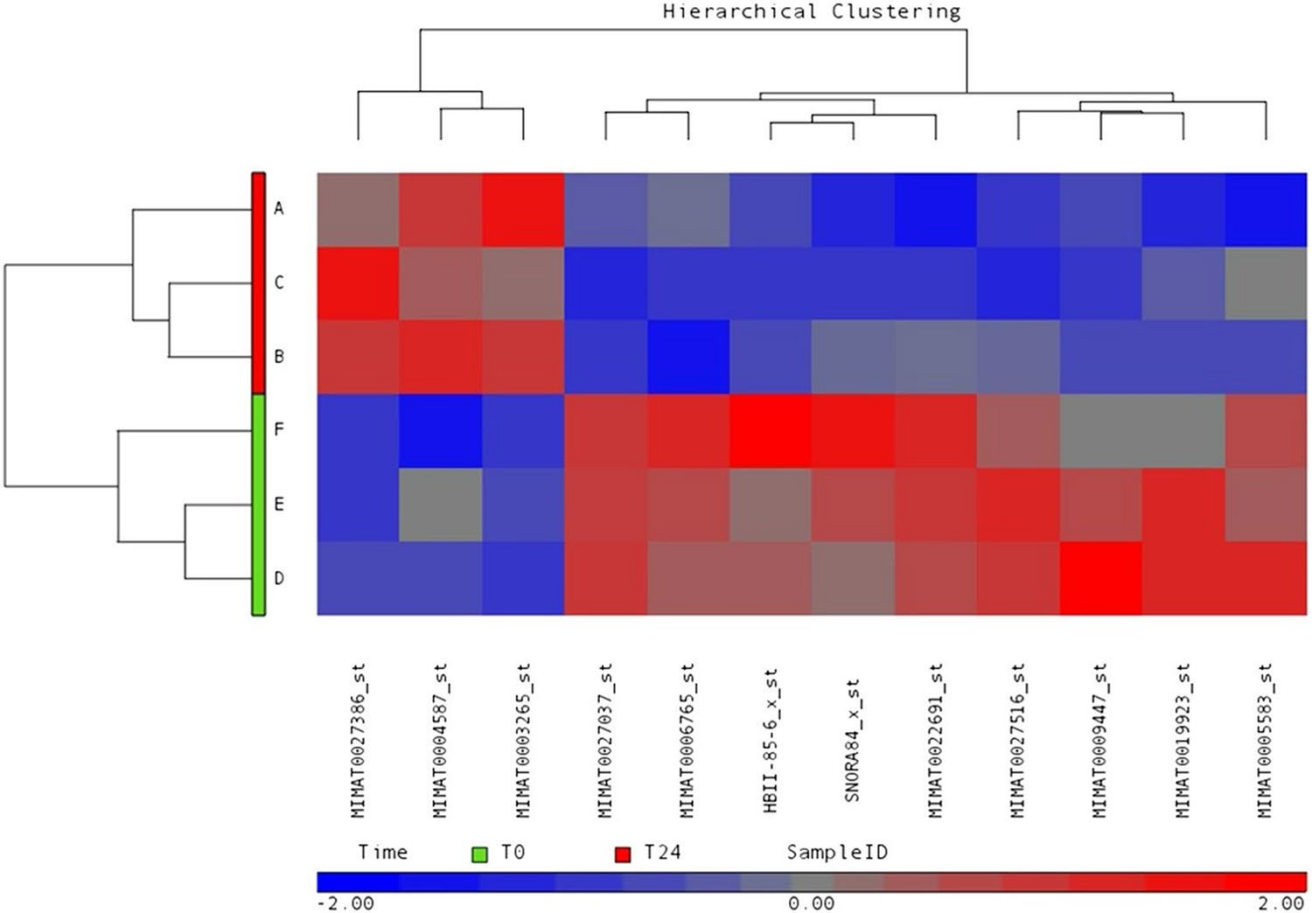
microRNA expression heat map. Differential miRNA expression in CVID serum patients after the first Ig replacement therapy (t24). The heat-map showing the differential expression pattern of miRNAs compared to CVID serum patients before the first Ig replacement therapy (t0). Blue represents low expression and red high expression. PARTEK Genomic Suite Software from Affymetrix was used with filtering fold change = 1.5 and p-value = 0.05.

**Figure 3:**
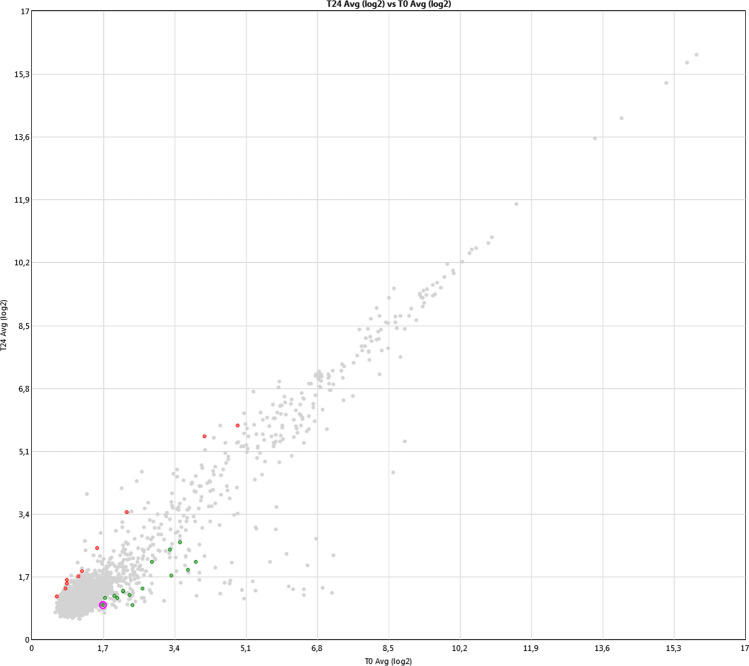
Scatter plot visualization of miRNAs identified using PARTEK Genomic Suite Software from Affymetrix. Filtering fold change = 1.5 and p-value = 0.05.

**Figure 4:**
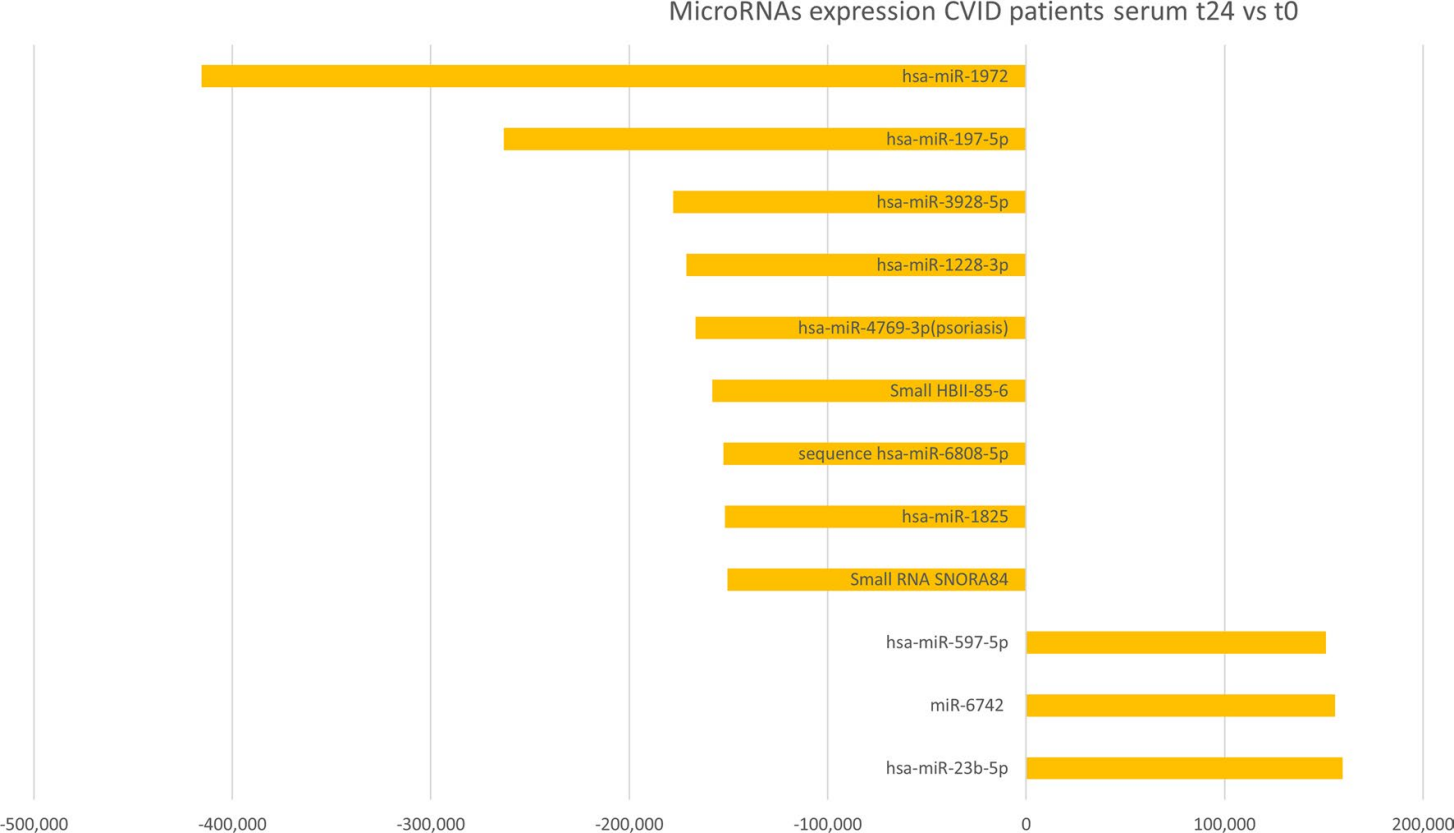
The barplot shows the most deregulated miRNAs and Small RNAs from microarray experiments in comparison between CVID serum patients after the first Ig replacement therapy (t24) vs CVID serum patients before the first Ig replacement therapy (t0). PARTEK Genomic Suite Software was used at filtering fold change = 1.5 and p-value = 0.05.

By microarray technology, it was possible to identify differentially expressed miRNAs in naïve CVID patients at basal T0 level (before any treatment) compared to the same CVID patients, 1 day after Ig infusion (T24). We used jointly a statistical test (t-test with cutoff set to 0.05, without applied FDR correction), together with a criterion based on the regulation entity (fold change). Experimental replicates (pool of CVID patients to create a biological triplicate for each experimental condition T0 and T24) were used. A fold change value of 1.5 was used to define a miRNA as differentially expressed. Two different bioinformatics tools from Affimetrix, PARTEK and TAC have been used here. TAC software uses hybridization background normalization and identification systems slightly different from those used by Partek. This is why the miRNA lists identified as differentially expressed with the same stringency parameters (fc1.5 and *p*value < 0.05) are in total number of 26 or greater, while with PARTEK there are 10 plus 2 Small nuclear RNAs (see Figs. 1,2,3,4).

### qRTPCR validation of microarray expression

**Figure 5:**
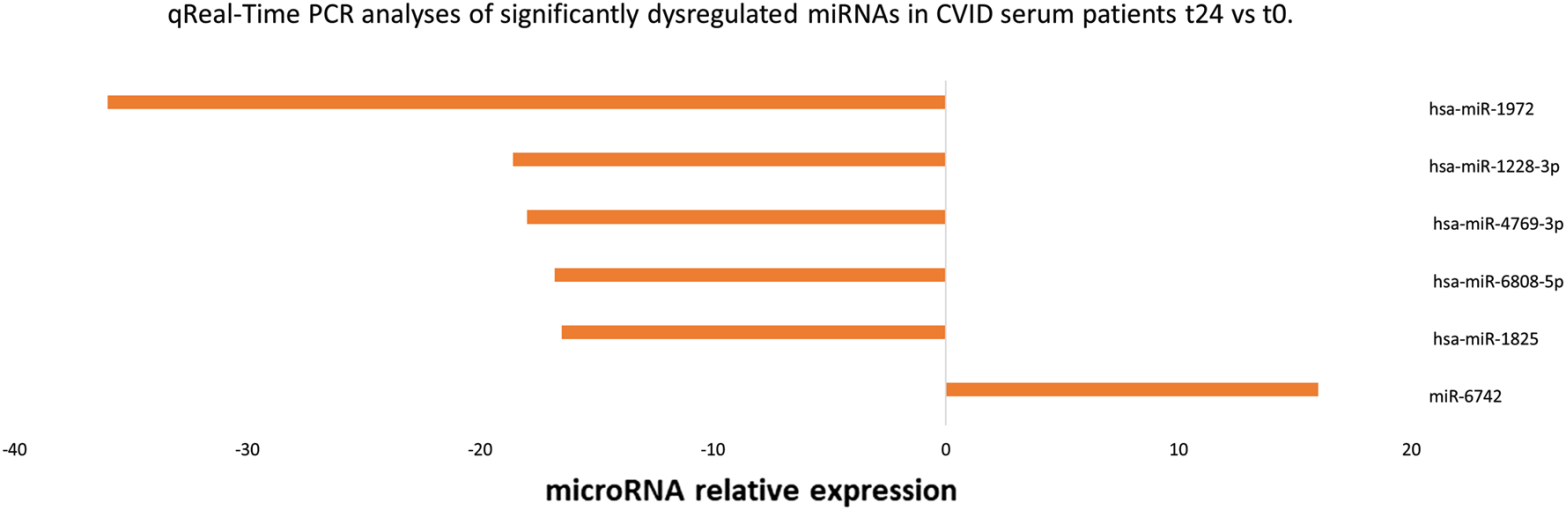
qRT-PCR validation of miRNAs: representative miRNAs were validated using qRT-PCR. Real-time PCR showing change in microRNA expression in CVID serum patients after the first Ig replacement therapy. Data are represented as the fold change in expression compared with control (CVID patients before the first Ig replacement therapy). *p* =  < 0.05.

Microarray expression results were validated by qPCR from serum and blood samples. miRNAs that showed a significant difference in expression (*p* < 0.05 and 1.5 fold change) between naïve CVID patients (T0) compared to the same patients 1 day after the first Ig replacement therapy (T24h) were selected for further Real-Time PCR analysis (see Fig. 5).

Among ten microRNAs, miR-6742, hsa-miR-1825, hsa-miR-4769-3p, hsa-miR-1228-3p, hsa-miR-1972, showed a significant expression between CVID patients (T0) compared to the same patients (T24h).

### Computational predictions of the putative mRNAs target

**Figure 6:**
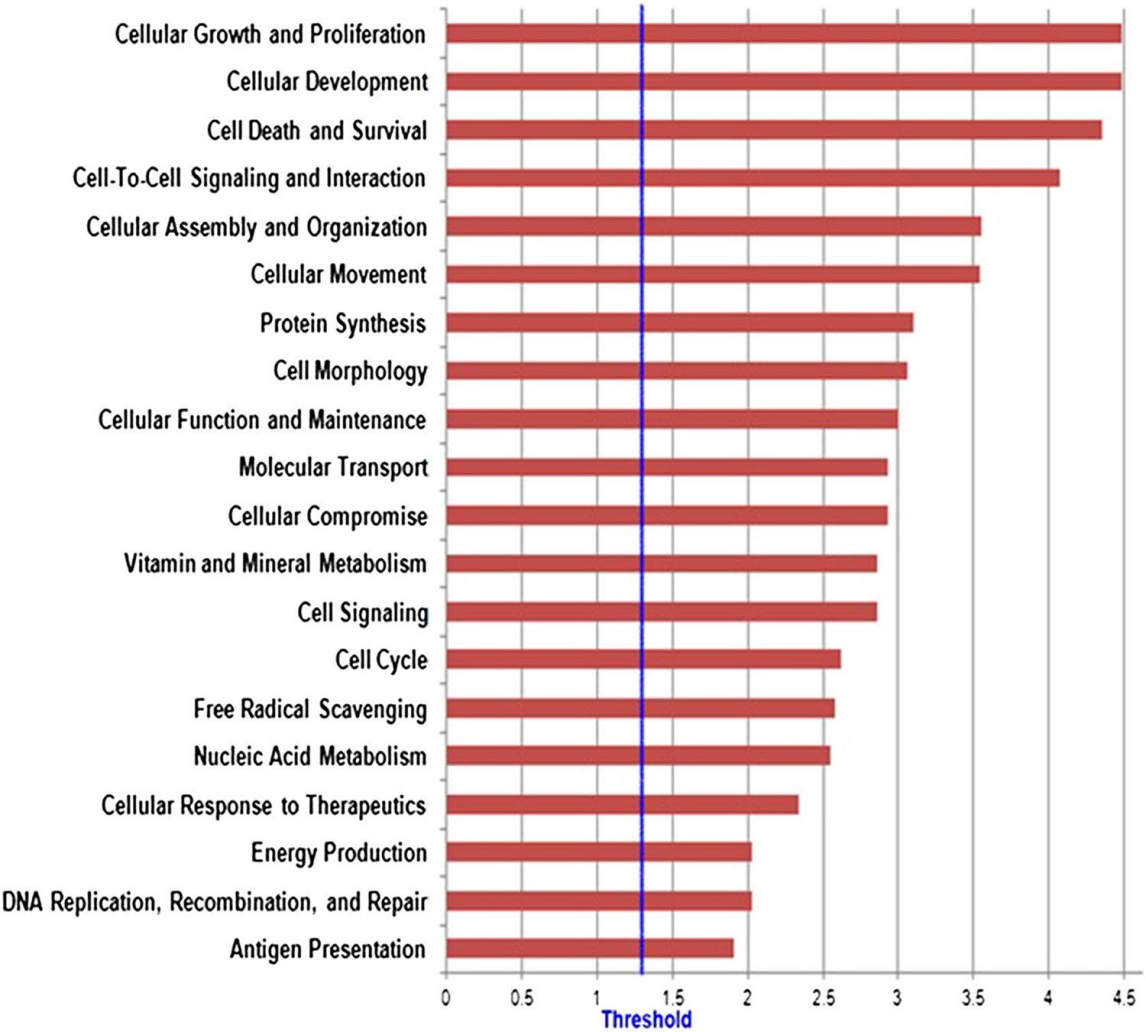
(red) Functional enrichment analysis by IPA (Ingenuity Pathway Analysis) of miRNA' mRNAs target. Only those functions with B-H p-value <  = 0.05 have been considered. The length of the bars is inversely proportional to -log10 (p-value). The blue line indicates the threshold of B-H p-value (Benjamini Hochberg) <  = 0.05.

**Figure 7:**
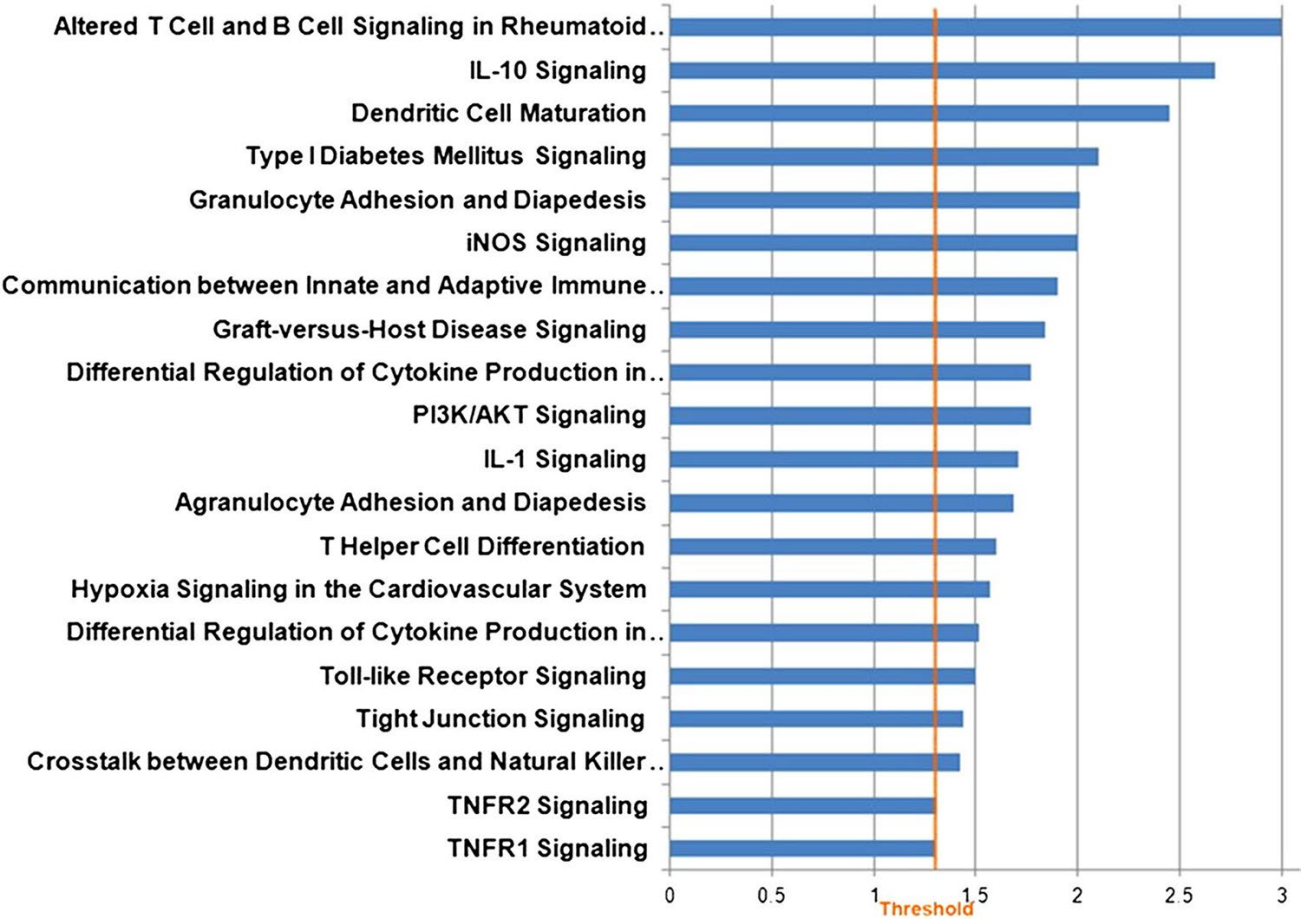
(blue) Canonical Pathway analysis by IPA (Ingenuity Pathway Analysis) of miRNA' mRNAs target. Only those pathways with B-H pvalue <  = 0.05 have been considered. The length of the bars is inversely proportional to -log10 (p-value). The orange line indicates the threshold of B-H p-value (Benjamini Hochberg) <  = 0.05.

MicroRNAs are particularly interesting for potential Target Genes, analyzed with the criterion that miRNAs can bind the 3′-UTR sequences of mRNAs. We explored putative miRNA target genes by searching them on three distinct web-accessible miRNA target databases, including TargetScan, PicTar, and miRDB. Numerous target mRNAs were identified using the results obtained by intersecting three different bioinformatics tools. In particular, we submitted the target genes obtained from this approach to the KEGG pathway and Gene Ontology tools both implemented in String database (Fig. 6, 7).

**Figure 8:**
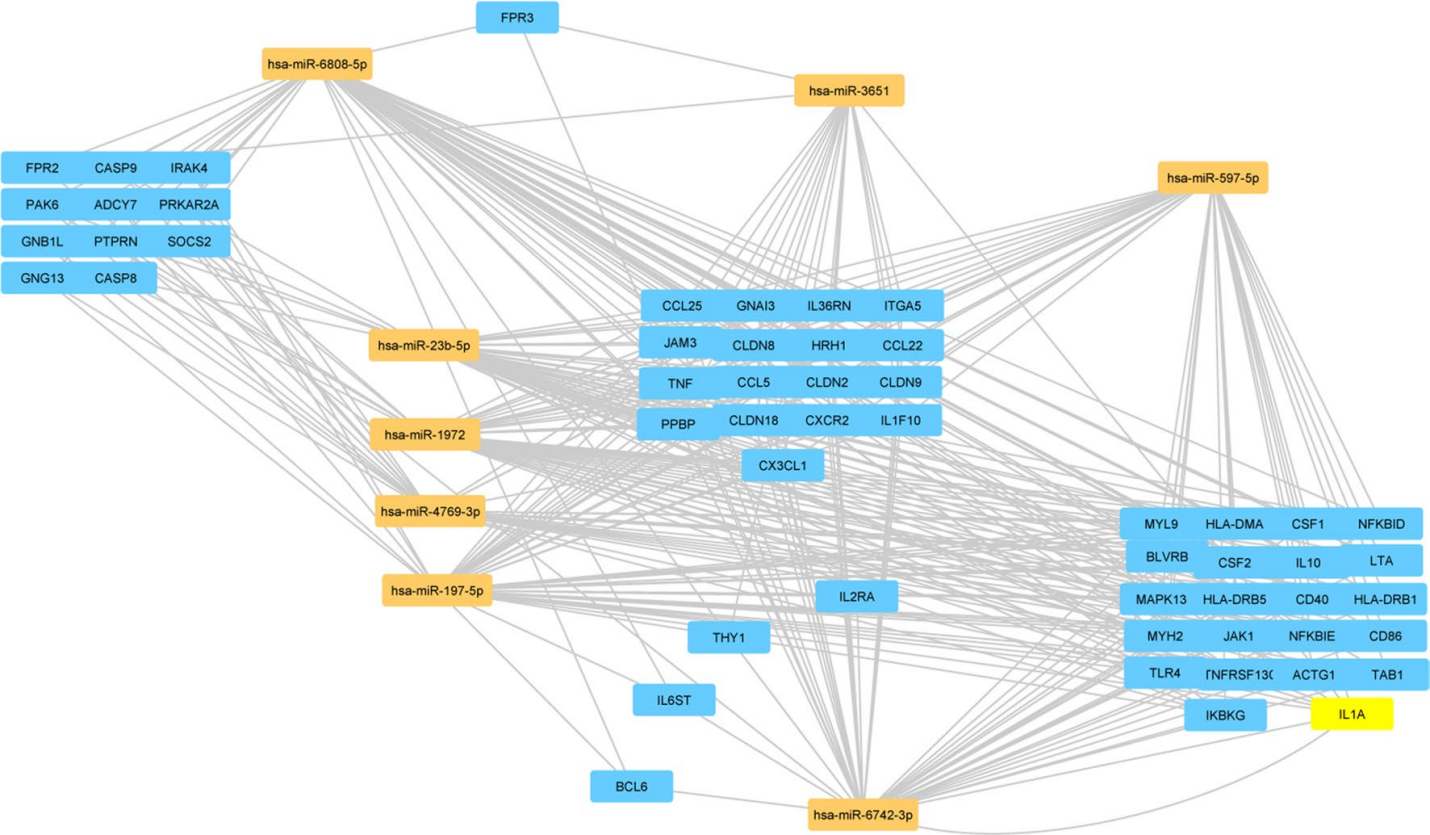
Combined molecular analysis in CVID serum patients before and after the first Ig replacement therapy (t24 vs t0). Functional annotations of target genes together with their miRNAs are visualized as a network workflow (Cytoscape 3.6.0).

Regarding the Gene Ontology analysis, as shown in Fig. 6, we obtained general terms enriched for the Molecular function. However, as shown in Ingenuity Canonical Pathway (Fig. 7), we observed a statistically significant enrichment of a subgroup of genes involved in immune-system pathways as TNFR, IL-1 and IL-10 signalling, cytokine production and regulation and communication between innate and adaptive Immune System. Those genes have also been associated with Type 1 diabetes mellitus signalling, an autoimmune disease. Figure 8 shows an example of the resulted molecular networks outputs that includes miRNAs isolated in this research (and target genes involved in the immune response functional categories.

In particular, in the Table 2 are listed all the pathways related to the immune system compartment in which this set of microRNA and mRNA target genes is involved.

**Table 2:** Ingenuity Canonical Pathways of miRNA’ mRNA target.

Ingenuity Canonical Pathways	-log(p-value)	Molecules	miRNAs
Altered T Cell and B Cell Signaling in Rheumatoid Arthritis	3	IL1A,IL10,IL1F10,TNFRSF13C,TLR4,HLA-DRB1,CD40,HLA-DMA,CSF1,LTA,IL36RN,CD86,CSF2,TNF,HLA-DRB5	hsa-miR-597-5p, hsa-miR-6742-3p,hsa-miR-197-5p,hsa-miR-6808-5p,hsa-miR-23b-5p,hsa-miR-1972
IL-10 Signaling	2.67	NFKBID,IKBKG,IL1A,JAK1,IL10,NFKBIE,IL36RN,BLVRB,MAPK13,IL1F10,TNF,TAB1	hsa-miR-597-5p, hsa-miR-6742-3p,hsa-miR-197-5p,hsa-miR-4769-3p
Type I Diabetes Mellitus Signaling	2.1	JAK1,NFKBIE,MAPK13,NFKBID,IKBKG,CASP9,HLA-DRB1,HLA-DMA,LTA,CD86,SOCS2,CASP8,TNF,PTPRN,HLA-DRB5	hsa-miR-4769-3p,hsa-miR-23b-5p,hsa-miR-6808-5p,hsa-miR-1972
Granulocyte Adhesion and Diapedesis	2.01	FPR3,IL1A,PPBP,CLDN18,FPR2,ITGA5,THY1,CCL22,IL1F10,CCL5,GNAI3,HRH1,CLDN8,CXCR2,JAM3,IL36RN,CCL25,CLDN2,CLDN9,CX3CL1,TNF	hsa-miR-6742-3p,hsa-miR-3651,hsa-miR-6808-5p
Differential Regulation of Cytokine Production in Intestinal Epithelial Cells by IL-17A and IL-17F	1.77	IL1A,IL10,CCL5,CSF2,TNF	hsa-miR-597-5p, hsa-miR-6742-3p,hsa-miR-197-5p,hsa-miR-6808-5p,hsa-miR-23b-5p
IL-1 Signaling	1.71	NFKBID,GNAI3,IKBKG,IL1A,NFKBIE,PRKAR2A,GNG13,MAPK13,GNB1L,ADCY7, TAB1,IRAK4NFKBID,GNAI3,IKBKG,IL1A,NFKBIE,PRKAR2A,GNG13,MAPK13,GNB1L,ADCY7, TAB1,IRAK4	hsa-miR-197-5p,hsa-miR-4769-3p,hsa-miR-6808-5p
Agranulocyte Adhesion and Diapedesis	1.69	IL1A,PPBP,CLDN18,ITGA5,CCL22,IL1F10,CCL5,MYL9,GNAI3,HRH1,MYH2,CLDN8,CXCR2,JAM3,IL36RN,CCL25,CLDN2,CLDN9,CX3CL1,ACTG1,TNF	hsa-miR-597-5p, hsa-miR-6742-3p,hsa-miR-197-5p,hsa-miR-6808-5p,hsa-miR-23b-5p,hsa-miR-1972,
T Helper Cell Differentiation	1.6	IL6ST,HLA-DRB1,CD40,HLA-DMA,IL10,CD86,IL2RA,BCL6,TNF,HLA-DRB5	hsa-miR-6808-5p,hsa-miR-197-5p,hsa-miR-6742-3p
Differential Regulation of Cytokine Production in Macrophages and T Helper Cells by IL-17A and IL-17F	1.52	IL10,CCL5,CSF2,TNF	hsa-miR-6742-3p,hsa-miR-23b-5p
TNFR2 Signaling	1.31	NFKBID,IKBKG,NFKBIE,LTA,TNF	hsa-miR-23b-5p,hsa-miR-4769-3p,hsa-miR-6808-5p
TNFR1 Signaling	1.3	NFKBID,IKBKG,CASP9,PAK6,NFKBIE,CASP8,TNF	hsa-miR-23b-5p,hsa-miR-4769-3p,hsa-miR-6808-5p,hsa-miR-1972

## Discussion

Our study is the first to identify miRNAs involved in Ig response pathways of CVID patients. Currently, the diagnosis and therapy of CVID is complex and expansive to conduct; therefore, the identification of non-invasive specific potential markers is necessary. On the other hand, the Ig replacement therapy represents the most value therapeutic approach for CIVD patients. In the current study, we used microarray assay and qPCR validation to screen miRNAs differentially expressed in serum from naïve patients with CVID, before (T0) and 24 h after (T1) the first IgG replacement therapy. In the clinical practice, patients are recalled at 24 h post-infusion as this is the optimum timing to observe IgG levels and/or adverse effects. In addition, we previously demonstrated^5^ that adiponectin levels are modulated by the Ig treatment specifically in CVID patients already at 24 h considering this cytokine as a biomarker for the replacement therapy in CVID patients. Finally, Quinti et al. demonstrated that an IgG dose higher than 6 mg/dL is effective in conferring immune protection; in our patients, at 24 h post-infusion, IgG levels were 6.92 ± 1.43 mg/dL indicating that 24 h is the timing to observe modification induced by Ig treatment and search for biomarkers^13^.

miRNAs have been extensively studied in many diseases, including immune disorders. Indeed, in the immune system, miRNAs have a wide range of significant functions, such as B cell proliferation, survival and maturation^13^. On the other hand, literature data demonstrated that the IgG therapy alters the expression of micro RNAs in different diseases both in vitro and in vivo^10–12^.

In the pathogenesis of CVID, in extensive genetic analysis, most patients do not have a monogenetic diagnosis however, familial aggregation of cases was reported^14^. Therefore, CVID might be considered an epigenetic phenomenon^15^.

The investigation of the clinical impact of microRNAs in human CVID is at a very initial point. In mice models, miR-142 has been found highly expressed in immune cells, and miR-142 mice knock-out models show phenotypic similarities to CVID with immunodeficiency, hypogammaglobulinaemia, and polyclonal lymphoproliferation^16^ supporting the significance of microRNAs in the regulation of B-cell functions. Furthermore, bic/miRNA-155-deficient mice display phenotypic similarities to CVID patients with adaptive immunodeficiency, hypogammaglobulinaemia, lung disease, and enteric inflammation^17^.

In our research, for the first time, we identified microRNAs in naïve CVID patients involved in the response to the therapy with Ig infusion. In particular, has-miR-6742, hsa-miR-1825, hsa-miR-4769-3p, hsa-miR-1228-3p, hsa-miR-1972, resulted modulated by Ig infusion in CVID patients. All of them are down-regulated except for miR-6742 that is up-regulated. Previous studies demonstrated an association between some alleles encoded in the MHC region and CVID; the presence of these alleles can influence disease susceptibility^18^. In this context, the different expression of miR-6742 (related to pathways involving Class I MHC mediated antigen processing and adaptative and Innate Immune System) could confirm the important role of the MHC genes in CVID^19^. In addition, miR-6742 has been associated with an autoimmune disease, Type 1 diabetes mellitus, strengthening its involvement in the immune system regulation^20^.

hsa-miR-1825, hsa-miR-4769-3p, hsa-miR-1228-3p, hsa-miR-1972 are all down-regulated after infusion with Ig in CVID patients. In accordance with our results, hsa-miR-1825 has been associated with immune disorders as systemic lupus erythematosus and rheumatoid arthritis where it is down-regulated^21^.

Recently, hsa-miR-4769-3p has also been found related to psoriasis^22^. Although additional studies are needed to clarify its biological functions in this pathology, it is clearly known that psoriasis has an immune basis, and that the immune system deregulation plays a central role in the development and severity. Interestingly, psoriasis has also been reported as an autoimmune manifestation in CVID patients^23^^.^

hsa-miR-1972 has also been involved in the immune disorders rheumatoid Arthritis and Type I Diabetes Mellitus; in particular, the hsa-miR-1972 is related to signaling pathways as altered T and B Cell Signaling, and TNFR1 signaling (Table 2). In Chronic myeloid leukemia has been shown that over expression of miR-1972 resulted in the cell cycle arrest at G2-M stage^24^. Earlier, it was proposed that global miRNA expression may reflect the state of cellular differentiation and miRNAs can prevent the cell division and drive the cellular differentiation^25^.

In conclusion, the identification of miRNAs modulated by Ig replacement therapy might help to understand the molecular mechanism of CVID etiopathogenesis. In addition, miRNAs could represent a valid target to develop potential biomarkers for the management of CVID patient therapy. Identification of the molecular cause underlying CVID is important due to the increasing availability of precision medical interventions^9,10^. Similarly, epigenetic therapies developed may also be utilized if epigenetic etiologies for CVID will be elucidated^11,12^. Many therapies are nowadays targeting microRNAs and some of them are already in clinical trials.

One limitation of this study is the absence of healthy controls as well as a different immunodeficiency population needing the Ig replacement therapy, to unequivocally relate the identified miRNAs to the therapy in CVID. Further studies are necessary to clarify the functional role of microRNAs isolated here in CVID patients and we speculated that in the future, epigenetic and genetic information could help guide more effective targeted therapeutic intervention in CVID.

## Materials and methods

### Recruitment of patients

Nine CVID treatment-naïve patients (six men and three women), diagnosed according to European Society for Immunodeficiencies diagnostic criteria of 2014, were recruited by the Division of Allergy and Clinical Immunology of the Department of Translational Medical Sciences, Università di Napoli “Federico II”. The height and weight of patients were measured using standard techniques and the BMI was calculated as body weight (kg) /height^2^ (m^2^). Blood samples (5 ml) were obtained (after 12 h of fasting) before and 24 h after the first Ig and replacement therapy (0.4 g/kg). In all patients, the levels of Ig G, IgA, Ig M were measured before and after Ig replacement. Biochemical parameters (Total Cholesterol, Triglycerides, Glucose, Total Proteins, Iron, Fibrinogen, CRP and ESR) were determined by standard enzymatic methods (Hitachi Modular, Roche, Mannheim, Germany) (see Table 1). The patients received Ig replacement therapy (IgVena 50 mg/ml by Kedrion S.p.A. at the dose of 0.4 g/kg/21 days). From the medical files of CVID patients, we recorded clinical history of recurrent infections, chronic diarrhea, bronchiectasis, autoimmune diseases (autoimmune hemolytic anemia, immune thrombocytopenia, neutropenia), polyclonal lymphoproliferation (splenomegaly, lymphadenopathy, and granulomatous disease), and malignancies^5^. Clinical phenotypes of patients were: 88.8% of patients have lymphocytic hyperplasia, 33.3% autoimmune cytopenias, 55.5% chronic enteropathy.

The research protocol was approved by the Ethics Committee of the School of Medicine, Università di Napoli “Federico II” and was conducted in accordance with the principles of the Helsinki II Declaration. Written informed consent was obtained from all participants.

### RNA extraction

The serum samples were extracted from the patients, centrifuged at 12,000 × g for 5 min at 4 °C and stored at -80 °C. Total RNA was extracted using Trizol reagent (Invitrogen, #15,596–026) method has been used for all samples in 1 h from drawing thus reducing RNA degradation. The RNA isolated with this protocol comes from all white cells, including polymorphonuclear leukocytes and mononuclear cells. RNA was isolated including an optional DNase digestion step. This standardized RNA isolation procedure guarantees high quality non-degraded RNA. RNA samples which were quality-checked by identification of 18S rRNA and 28S rRNA peaks via the Agilent 2100 Bioanalyzer platform (Agilent Technologies)^26^.

### Target preparation

For miRNA expression analysis, the Affymetrix Genechip miRNA 4.0 array (Affymetrix; Thermo Fisher Scientific, Inc.) was used according to the manufacturer's instructions. Briefly, 300 ng of total RNA was poly-A tailed and biotin-labeled using the FlashTag Biotin HSR RNA Labeling Kit (Affymetrix, Genisphere, Thermo Fisher Scientific, Inc). As suggested by manufacturer’s protocol, Poly-A RNA Controls were added to each sample, to allow GeneChip probe array users to assess the overall success of the assay^26^.

### Microarray hybridization

Hybridization of each target was performed using the GeneChip Hybridization, Wash and Stain Kit (Affymetrix; Thermo Fisher Scientific, Inc)^26^. It contains mix for target dilution, DMSO at a final concentration of 9,7% and pre-mixed biotin-labelled control oligo B2 and bioB, bioC, bioD and cre controls at a final concentration of 50 pM, 1.5 pM, 5 pM, 25 pM and 100 pM, respectively. Targets were diluted in hybridization buffer, denatured at 99 °C for 5 min, incubated at 45 °C for 5 min and centrifuged at maximum speed for 1 min prior to introduction into the GeneChip cartridge. A single GeneChip miRNA 4.0 was then hybridized with each biotin-labeled target.

Hybridizations were performed for 17 h at 48 °C in a rotisserie oven (60 RPM). GeneChip cartridges were washed and stained with GeneChip Hybridization, Wash and Stain Kit in the Affymetrix Fluidics Station 450 following the FS450_0002 standard protocol, including the following steps. (1) (wash) 10 cycles of 2 mixes/cycle with Wash Buffer A at 30 °C; (2) (wash) 6 cycles of 15 mixes/cycle with Wash Buffer B at 50 °C; (3) stain of the probe array for 5 min in SAPE solution at 35 °C; (4) (wash) 10 cycles of 4 mixes/cycle with Wash Buffer A at 30 °C; (5) stain of the probe array for 5 min in antibody solution at 35 °C; (6) stain of the probe array for 5 min in SAPE solution at 35 °C; (7) (final wash) 15 cycles of 4 mixes/cycle with Wash Buffer A at 35 °C; (8) fill the probe array with Array Holding buffer.

### Image acquisition, processing and bioinformatic analysis

GeneChip arrays were scanned using an Affymetrix GeneChip Scanner3000 7G using default parameters. Affymetrix GeneChip Command Console software (AGCC) was used to acquire GeneChip images and generate .DAT and .CEL files, which were used for subsequent analysis with proprietary software ^24^. Data analysis has been performed using Partek Genomics Suite Software from Affimetryx and, to validate the results, Transcriptome Analysis Console (TAC) Software from Affimetryx with filtering fold change = 1.5 and p-value = 0.05.

### Reverse transcription-quantitative polymerase chain reaction (RT-qPCR)

cDNA was obtained by using oligo-dT primers or stem-loop reverse transcriptase (RT) primers, respectively. RNU6B was used as controls for microRNAs. Real-Time qPCR was performed under the following conditions: 94 °C for 4 min followed by 40 cycles at 94 °C for 1 min, 56 °C for 1 min and 72 °C for 1 min. Relative expression levels of hsa-miR-6742, hsa-miR-1825, hsa-miR-4769-3p, hsa-miR-1228-3p, hsa-miR-1972 were calculated using the 2^-ΔΔCt^ method^26^. Data were expressed as the mean ± standard deviation. A two-tailed Student's *t*-test was performed for group comparisons and *p* < 0.05 was considered statistically significant.

### MiRNA target prediction

The target mRNAs that have the potential binding sites for individual miRNAs were identified by searching them on public databases endowed with prediction algorithms, such as TargetScan (https://targetscan.org), PicTar (https://pictar.mdc-berlin.de), miRBase (https://www.mirbase.org), TarBase (https://microrna.gr/tarbase) and Miranda (https://microrna.sanger.ac.uk/sequences)27.

We considered potential significant targets those found significant in both methods, as previously suggested^27^. String database was used to build the Protein–Protein Interaction (PPI) network, and to perform Gene Ontology and functional annotation^28^.
